# IL27 gene expression distinguishes multisystem inflammatory syndrome in children from febrile illness in a South African cohort

**DOI:** 10.3389/fimmu.2022.992022

**Published:** 2022-09-06

**Authors:** Timothy F. Spracklen, Simon C. Mendelsohn, Claire Butters, Heidi Facey-Thomas, Raphaella Stander, Debbie Abrahams, Mzwandile Erasmus, Richard Baguma, Jonathan Day, Christiaan Scott, Liesl J. Zühlke, George Kassiotis, Thomas J. Scriba, Kate Webb

**Affiliations:** ^1^ Department of Paediatrics and Child Health, University of Cape Town, Cape Town, South Africa; ^2^ Cape Heart Institute, University of Cape Town, Cape Town, South Africa; ^3^ South African Tuberculosis Vaccine Initiative, Institute of Infectious Disease and Molecular Medicine, University of Cape Town, Cape Town, South Africa; ^4^ Division of Immunology, Institute of Infectious Disease and Molecular Medicine, University of Cape Town, Cape Town, South Africa; ^5^ South African Medical Research Council, Cape Town, South Africa; ^6^ Retroviral Immunology Laboratory, The Francis Crick Institute, London, United Kingdom; ^7^ Department of Infectious Disease, St Mary’s Hospital, Imperial College, London, United Kingdom; ^8^ Crick African Network, The Francis Crick Institute, London, United Kingdom

**Keywords:** COVID-19, multisystem inflammatory syndrome, children, South Africa, SARS-CoV-2

## Abstract

**Introduction:**

Multisystem inflammatory syndrome in children (MIS-C) is a severe acute inflammatory reaction to SARS-CoV-2 infection in children. There is a lack of data describing differential expression of immune genes in MIS-C compared to healthy children or those with other inflammatory conditions and how expression changes over time. In this study, we investigated expression of immune-related genes in South African MIS-C patients and controls.

**Methods:**

The cohort included 30 pre-treatment MIS-C cases and 54 healthy non-inflammatory paediatric controls. Other controls included 34 patients with juvenile systemic lupus erythematosus, Kawasaki disease or other inflammatory conditions. Longitudinal post-treatment MIS-C specimens were available at various timepoints. Expression of 80 immune-related genes was determined by real-time quantitative PCR.

**Results:**

A total of 29 differentially expressed genes were identified in pre-treatment MIS-C compared to healthy controls. Up-regulated genes were found to be overrepresented in innate immune pathways including interleukin-1 processing and pyroptosis. Post-treatment follow-up data were available for up to 1,200 hours after first treatment. All down-regulated genes and 17/18 up-regulated genes resolved to normal levels in the timeframe, and all patients clinically recovered. When comparing MIS-C to other febrile conditions, only *IL27* expression could differentiate these two groups with high sensitivity and specificity.

**Conclusions:**

These data indicate a unique 29-gene signature of MIS-C in South African children. The up-regulation of interleukin-1 and pyroptosis pathway genes highlights the role of the innate immune system in MIS-C. IL-27 is a potent anti-inflammatory and antiviral cytokine that may distinguish MIS-C from other conditions in our setting.

## Introduction

Multisystem inflammatory syndrome in children (MIS-C) is a severe acute inflammatory reaction to SARS-CoV-2 infection in children. MIS-C patients typically present with hyperinflammation, fever, gastrointestinal symptoms, myocardial dysfunction and other variable systemic symptoms requiring hospitalisation (1, 2), and are often healthy before SARS-CoV-2 exposure (3). Early descriptions of MIS-C noted similarities with Kawasaki disease (KD) and toxic shock syndrome, although clinical, immunological and laboratory distinctions between these conditions have been reported (4–10). Data on MIS-C in Africa is lacking, despite black African ethnicity being a risk factor for MIS-C with worse outcomes than other population groups (11–13). MIS-C has been described in Africa albeit in limited reports (14–16), with an estimated incidence of 22/100,000 SARS-CoV-2 exposures in our setting (16).

MIS-C patients have high serum titres of neutralising IgG SARS-CoV-2 antibodies, suggesting a post-infectious aetiology (2). Although the immunological profile of MIS-C is not yet completely understood, investigations have reported elevated levels of several inflammatory and migratory cytokines, presence of autoantibodies, and immune cell repertoires that could be distinguished from paediatric or adult COVID-19 patients and KD (5, 9, 17–20). Polyclonal *TRBV11-2* T cell enrichment suggests superantigen stimulation may underly MIS-C pathogenesis (19, 21).

There is a lack of sufficiently powered studies of the expression of immune genes in children with MIS-C over time and compared to controls (both healthy and inflammatory). A whole blood RNA sequencing analysis of 8 MIS-C patients and 13 controls reported an MIS-C transcriptomic signature characterised by 2,043 differentially expressed genes (DEGs) (22); co-expression network analysis of this signature revealed significant downregulation of CD8+ T cells and natural killer cells. A separate RNA-sequencing experiment compared 12 MIS-C patients to 13 febrile controls and identified a hyperinflammatory cluster of children who were chiefly severely affected by MIS-C (9). This cluster demonstrated increased neutrophils and inflammatory markers, and a reduction of adaptive immune cells. Milder cases tended to cluster more strongly with febrile controls in this analysis.

In the present study, we investigated expression of immune-related genes in a cohort of South African MIS-C patients and controls.

## Methods

### Participant recruitment and follow-up

Participants were recruited from Red Cross War Memorial Children’s Hospital in Cape Town, South Africa, between June 2020 and June 2021. Children with confirmed MIS-C as per WHO criteria (23) were recruited with consent. Included were MIS-C patients sampled before treatment with intravenous immunoglobulin (IVIG) or steroid (pre-treatment) as well as children who were suspected of having MIS-C but with an ultimate alternate diagnosis (inflammatory controls). Healthy control children were recruited before elective surgery for non-inflammatory indications (e.g. circumcision, strabismus repair) as previously described (16). Other controls included patients who met the criteria for diagnosis with juvenile systemic lupus erythematosus (JSLE) (24) and KD (25). For patients who met criteria for both KD and MIS-C, those who had no evidence of exposure to SARS-CoV-2 (seronegative for SARS-CoV-2 antibodies and PCR negative) were classified as KD. The clinical profiles and treatment of this Cape Town MIS-C cohort have been described previously (16).

Where possible, patients were followed up post-treatment. Longitudinal post-treatment MIS-C specimens were available at various timepoints for IVIG alone (n = 10) or IVIG and methylprednisolone (n = 30). Longitudinal treatment data were not available for the JSLE, KD and inflammatory control group.

The protocol was approved by the Human Research Ethics Committee of the Faculty of Health Sciences at the University of Cape Town (HREC 599/2020) and all research was performed in accordance with relevant regulations. Parents or legal guardians provided written informed consent in their language of choice, and where possible, participants provided assent.

### RNA extraction and gene expression analysis

RNA was extracted from PAXgene Blood RNA tubes using the PAXgene Blood RNA kit and used as the template for cDNA synthesis using EpiScript reverse transcriptase. Transcripts of interest were pre-amplified using a pool of 96 TaqMan primer-probe assays (**Supplementary Table S1**). Pre-amplified cDNA, along with an internal positive control and a no-template (water) control, and the 96 primer-probe assays, were loaded into a microfluidic 96.96 Gene Expression Integrated Fluidic Circuit (Fluidigm) and gene expression of individual transcripts was then quantified by qRT-PCR performed on a BioMark HD (Fluidigm) instrument. The following parameters were applied for extracting cycle threshold (Ct) values: Linear (Derivative) baseline correction, Quality Threshold of 0.3 and Auto (Global) for Ct Threshold Method using Fluidigm software version 4.5.2. Only primer/probe assays with a ≥ 70% pass rate across all samples, and samples with a ≥ 90% assay pass rate were retained for downstream analysis. All gene expression data were generated by blinded laboratory personnel; only once gene expression data were locked down were group allocations unblinded for analyses.

### Statistical analysis

Quality control (including reproducibility against an internal positive control sample) and analysis of gene expression Ct data was performed as previously described (26) in R version 4.0.3. Delta Ct values were computed for each transcript relative to the mean of three reference transcripts (*ACTR3*, *CDC42* and *USF2* selected as previously described (27)).

Gene expression levels were compared using two-tailed Mann Whitney U tests and corrected for multiple comparisons using the Holm-Bonferroni method. Statistical outliers, defined as values beyond 1.5 interquartile ranges from the first and third quartiles, were removed from the pairwise analysis. Unsupervised hierarchical clustering was performed using the pheatmap R package with centering and scaling of variables. For principal component analysis (PCA), random-forest-based imputation of missing delta Ct values was performed using the missForest package (28) in R with 100 trees in each forest. PCA was done using the base R prcomp function with zero centering and scaling of variables. Receiver operating characteristic analysis was performed using the pROC package (29). Significant associations between MIS-C and healthy controls were corrected for neutrophil-lymphocyte ratio (NLR) using binomial logistic regression in base R, in which NLR data were available for 17 MIS-C participants and randomly estimated within the healthy expected ranges for the 54 healthy controls. P-values < 0.05 were considered statistically significant in all analyses.

## Results

### Study participants

The study cohort (**Table 1**) consisted of 30 pre-treatment MIS-C patients, 54 healthy non-inflammatory controls (15 with prior SARS-CoV-2 exposure, 37 without SARS-CoV-2 exposure, and 2 with unknown exposure at enrollment), 19 inflammatory controls with diverse diagnoses (**Supplementary Table S2**), as well as 8 patients with KD and 7 patients with JSLE. There were significantly more patients of black African ancestry in the MIS-C group compared to the inflammatory and healthy controls (p = 0.019). All the MIS-C patients included in this study were hospitalised, and all presented with fever and tachycardia (**Supplementary Table S3**). Other common presenting symptoms were rash, conjunctivitis and abdominal pain. Only three children (10%) had a positive PCR SARS-CoV-2 test on admission. During their hospital stay, 9 patients were admitted to ICU, but all 30 participants recovered during the study period.

**Table 1:** Baseline characteristics of the MIS-C and control qPCR cohorts.

	MIS-C (n = 30)	Inflammatory controls (n = 19)	Healthy controls (n = 54)	KD (n = 8)	JSLE (n = 7)
**Age (years)**					
Median	5.0	5.8	5.2	2.0	12.1
IQR (Q1; Q3)	7.0 (2.8; 9.8)	5.9 (4.0; 9.9)	7.0 (2.8; 9.8)	6.5 (0.7; 7.3)	0.5 (12.0; 12.5)
**Sex**					
Male (%)	14 (46.7%)	15 (78.9%)	32 (59.3%)	4 (50.0%)	1 (14.3%)
Female (%)	16 (53.3%)	4 (21.1%)	22 (40.7%)	4 (50.0%)	6 (85.7%)
**Ancestry**					
Black African (%)	19 (63.3%)	9 (47.4%)	18 (33.3%)	7 (87.5%)	2 (28.6%)
Cape Mixed (%)	10 (33.3%)	10 (52.6%)	36 (67.7%)	1 (12.5%)	5 (71.4%)
Not reported (%)	1 (3.3%)	0	0	0	0
**Comorbidities**					
Obesity/overweight (%)	3 (10.0%)	1 (5.3%)	0	0	0
Bone disorder (%)	2 (6.7%)	0	0	0	0
Dermatological (%)	1 (3.3%)	0	0	0	0
Infectious (%)	1 (3.3%)	2 (10.5%)	0	1 (12.5%)	0
Developmental (%)	0	1 (5.3%)	0	1 (12.5%)	0
None/not reported (%)	23 (76.7%)	15 (78.9%)	54 (100.0%)	6 (75.0%)	7 (100.0%)
Epidemiological wave*					
During or after first wave	21 (70.0%)	9 (47.4%)	34 (63.0%)	5 (62.5%)	1 (14.3%)
During or after second wave	7 (23.3%)	8 (42.1%)	19 (35.2%)	0	4 (57.1%)
During third wave	2 (6.7%)	2 (10.5%)	1 (1.8%)	3 (37.5%)	2 (28.6%)

*Epidemiological waves defined as per South African National Institute for Communicable Diseases criteria for the Western Cape province of South Africa.

KD, Kawasaki disease; JSLE, juvenile systemic lupus erythematosus; IQR, interquartile range; MIS-C, multisystem inflammatory syndrome in children.

### Unique 29-gene expression signature for MIS-C

**Figure 1:**
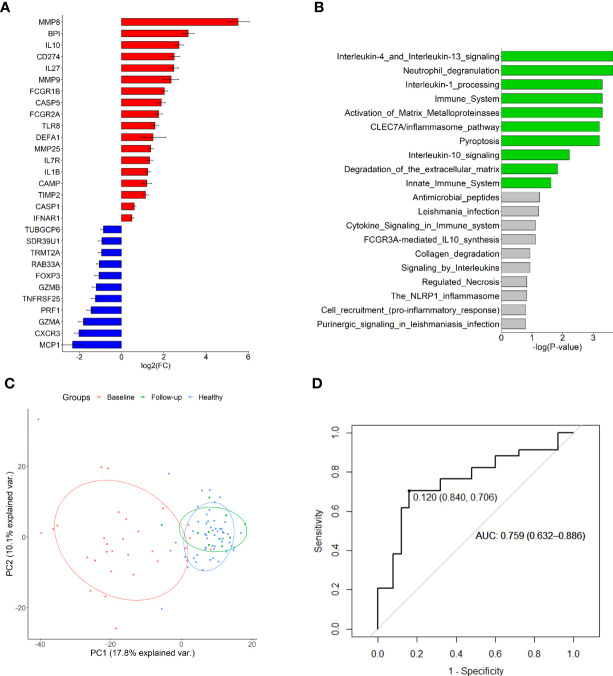
Differentially expressed genes in MIS-C. **(A)** Up-and down-regulated genes compared to healthy controls and **(B)** overrepresented Reactome pathways in the up-regulated gene set. Green bars indicate those with FDR < 0.05. **(C)** Principal component analysis of all investigated gene expression values in healthy controls and MIS-C at baseline and follow-up (mean 555.5 days). Plotted are principal components 1 and 2. **(D)** Receiver operating characteristic curve of *IL27* expression discriminating MIS-C from other febrile conditions.

Pairwise analysis of pre-treatment RNA expression across all experimental groups revealed 29 DEGs in MIS-C compared to healthy controls (**Figure 1A**; **Supplementary Table S4**). Reactome analysis of the 18 up-regulated genes in MIS-C revealed overrepresentation of many innate immune pathways including neutrophil degranulation, interleukin (IL)-1 processing, IL-4, -10 and -13 signalling, and pyroptosis in MIS-C (**Figure 1B**). There was no discernible enrichment of pathways in the down-regulated gene set.

Because of the inherent neutrophilia that is characteristic of MIS-C, and the linear relationships between expression of many of the investigated genes with neutrophils (**Supplementary Figure S1**), DEG associations were also calculated using available NLR data for 17 MIS-C participants and estimated normal NLRs for the 54 healthy controls. After adjustment for NLR, 11 DEGs remained significantly associated with MIS-C (*BPI*, *CAMP*, *CD274*, *CXCR3*, *DEFA1*, *FCGR1B*, *IL1B*, *IL10*, *IL27*, *MMP8* and *MMP9*) (**Supplementary Table S5**).

### Temporal RNA expression analysis reveals gene dysregulation and recovery in MIS-C

**Figure 2:**
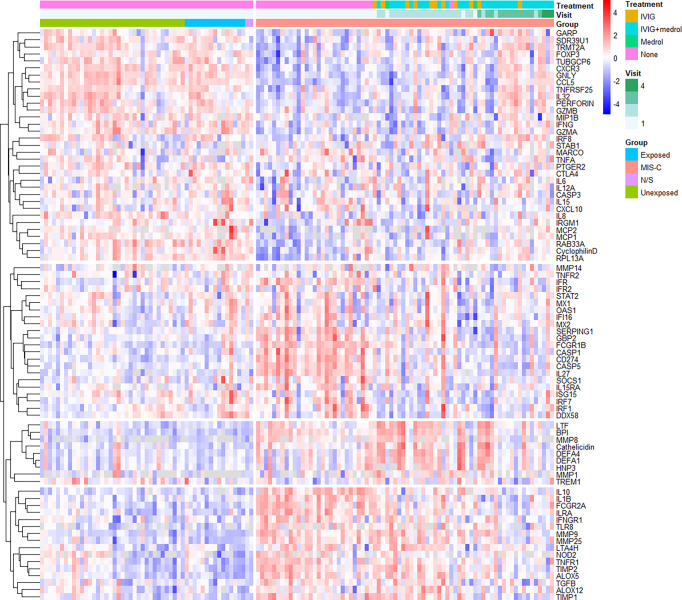
Unsupervised hierarchical clustering of gene expression. Samples are grouped by type (MIS-C, SARS-CoV-2 exposed or unexposed healthy controls or healthy controls for whom SARS-CoV-2 exposure was not stated [N/S]) and longitudinally in the case of MIS-C (up to three follow-up visits). The hierarchical tree is cut into four distinct clades: group 1 (top-most), genes that appeared down-regulated compared to controls at baseline; group 2 (second from top), genes that were up-regulated but quickly returned to normal after treatment; group 3 (second from bottom), genes that appeared to be induced by treatment; and group 4 (bottom-most), up-regulated genes that slowly returned to normal over the study period. Healthy controls include those with no recorded exposure to SARS-CoV-2 (unexposed), those with recorded exposure (exposed), and uncertain exposure (N/S). *IVIG – intravenous immunoglobulin; N/S – not stated*.

Unsupervised clustering analysis of the expression data revealed several changes in gene expression, both between MIS-C and healthy controls, and in MIS-C patients over time (**Figure 2**), from which four gene clades could be resolved. These included (1) genes that appeared down-regulated compared to controls at baseline, (2) genes that were up-regulated but quickly returned to normal after treatment, (3) genes that appeared to be induced by treatment, and (4) up-regulated genes that slowly returned to normal over the study period.

Group 1 contained many genes involved in chemotaxis and chemokine-mediated signalling, B cell homeostasis, differentiation or regulation, cytolysis and the response to interferon (IFN)-gamma. Group 2 was characterised by innate immune response genes, viral defence genes, negative regulators of type I IFN signalling, and positive regulators of IFN beta production. Group 3 contained many genes involved in antimicrobial humoral responses and innate mucosal immune responses, while group 4 contained genes involved in B cell proliferation, and negative regulation of inflammation.

### Gene expression largely recovers by follow-up

Analysis of longitudinal post-treatment specimens (n = 40) indicated an improvement of all down-regulated and all but one up-regulated gene over the follow-up period of up to 1,200 hours (**Supplementary Figures S2–5**). Only *MMP9* appeared to remain elevated over the follow-up period. Non-inflammatory healthy controls and MIS-C participants could be resolved through PCA of the baseline pre-treatment RNA expression data (**Figure 1C**), although principal components 1 and 2 could only account for < 30% of variation between the groups. PCA of longitudinal specimens demonstrated a recovery in gene expression by follow-up (mean follow-up period of 555.5 hours).

### *IL27* expression discriminates MIS-C from other febrile conditions

Pairwise comparison of the expression profiles of 30 baseline MIS-C patients to 34 participants with other febrile conditions (inflammatory controls, KD and JSLE) revealed *IL27* as the only DEG after correction for multiple testing (p = 0.00056; **Supplementary Table S6**). *IL27* expression could reasonably discriminate MIS-C from other conditions (**Figure 1D**), with an AUC of 0.759 (95% CI 0.632-0.886) and a cut-off expression value of 0.12 yielding 84% specificity and 71% sensitivity. Inclusion of other nominally significant DEGs in a polygenic score did not substantially increase predictive capacity (**Supplementary Figure S6**).

## Discussion

In this study, we characterised a 29-gene signature of transcripts differentially expressed in MIS-C compared to healthy control children, including up-regulation of genes involved in innate immune pathways such as IL-1, IL-4, IL-13, inflammasomes and pyroptosis. Of these genes, *IL27* expression alone could differentiate MIS-C from other febrile patients presenting to our hospital. Our data also demonstrate recovery of almost all DEGs to normal levels by follow-up.

The up-regulation of IL-1, IL-4 and IL-13 pathways in MIS-C is in accordance with previous reports in which these cytokines have been elevated in MIS-C serum (17, 30–32). IL-1 blockade has been used in the treatment of MIS-C and KD, and recently anti-IL-1Ra autoantibodies have been described in over 60% of MIS-C patients while absent in healthy controls and patients with COVID-19 (33). Cytokine profiling of MIS-C patients found that elevation of IL-4 and IL-13 could distinguish MIS-C from other hyperinflammatory conditions (31). Both are T helper type 2 inflammatory cytokines, and while neither was directly quantified in our assay, the up-regulation of genes in these pathways supports the roles of IL-4 and IL-13 in MIS-C pathogenesis, or may form part of the response to the hyperinflammation that is typical of this condition.

Inflammasome activation has also been implicated in the development of MIS-C, with increased RNA expression of several inflammasome-related genes in MIS-C and KD (34) – including *IL1B*, *CASP1* and *CASP5*, all of which were up-regulated in our cohort (**Figure 1A**). Inflammasomes were further implicated in SARS-CoV-2 pathogenesis when elevated levels of secretory phospholipase 2 were reported in both severe adult COVID-19 and acute MIS-C, returning to normal levels on recovery (35). It is known that SARS-CoV-2 infection triggers inflammasomes to release proinflammatory cytokines, with benefits of moderate inflammasome activation including antimicrobial responses, tissue repair, and induction of B and T cell responses (36). In contrast, excessive activation can lead to hyperinflammation and tissue damage (36), and may underly the similar inflammatory features seen in MIS-C. In addition to cytokine release, the caspases (including CASP1, up-regulated in this study) released by inflammasomes can cleave and activate gasdermin D to initiate pyroptosis. Pyroptosis is an inflammatory and lytic form of programmed cell death that has been implicated in triggering the cytokine storm in SARS-CoV-2 infected lung tissue and macrophages, contributing to hyperinflammation of the lungs (37, 38). Circulating plasma markers of pyroptosis were increased in COVID-19 compared to healthy controls and were reported to correlate with disease severity (39, 40); the authors suggested that SARS-CoV-2 uptake by monocytes and macrophages may trigger pyroptosis and lead to systemic inflammation in COVID-19 (39). While pyroptosis has not, to our knowledge, been reported in MIS-C to date, our data suggest that pyroptosis may also contribute to the systemic hyperinflammation in paediatric post-infectious SARS-CoV-2 responses.

The role of IL-27 in MIS-C is less well understood. Recently IL-27 was reported as one of several cytokines to be significantly up-regulated in MIS-C compared to febrile controls (41), although IL-27 was one of the most markedly elevated markers in MIS-C. While this observation was limited to seven MIS-C patients, it does corroborate the findings of our RNA study at the cytokine level. IL-27 is an anti-inflammatory cytokine that activates Th1 cells and natural killer cells, inhibits Th2 and Th17 cells, and stimulates release of IL-10 by T cells, and has been reported as a reliable predictor of adverse outcomes and mortality in COVID-19 (42–45). IL-27 also has potent antiviral effects against influenza, hepatitis B and C viruses, human immunodeficiency virus and others, by stimulating IFNγ production by CD8+ T cells and modulating innate responses (46). Marked elevation of IL-27 was reported in a single case of mRNA-1273 COVID-19 vaccine-related myocarditis, with more moderate increases in IL-27 in recently vaccinated control individuals (incidentally, inflammasome activation was also implicated in the vaccine-related myocarditis) (47). Indeed, IL-27 production by B cells has been shown to contribute to cytotoxic CD8 T cell responses to SARS-CoV-2 subunit vaccines, suggesting the importance of this cytokine in the viral response to SARS-CoV-2 (48, 49). Additionally, blockade of IL-27 was reported to improve mortality in mouse models of endotoxin- and sepsis-induced cytokine storms (50).

All genes except *MMP9* returned to normal levels by follow-up; *MMP9* was the only DEG that appeared to remain elevated within the study period. Increased MMP9, an elastolytic protease with both pro- and anti-inflammatory effects, has been implicated in several disorders including cardiovascular diseases (atherosclerosis, aortic aneurysm, myocardial infarction, and vascular inflammation (51)) and KD where it may play a role in development of coronary artery aneurysms (52–54). Although limited by the small number of follow-up samples in this study, this sustained elevation of *MMP9* in MIS-C may warrant further investigation due to the potential implications on cardiovascular inflammation and diseases.

Notably, *IL6* expression was not increased in this South African cohort compared to healthy controls, despite several prior studies demonstrating elevation of this cytokine in MIS-C (10, 17, 19, 55–61) or its association with MIS-C severity (62–66). In fact, although not statistically significant, *IL6* expression clustered into clade 1 (**Figure 2**): genes that appeared down-regulated in our cohort compared to healthy controls. This may be because RNA was quantified here rather than cytokines themselves, reflecting a limitation of the study. However, a recent comparison of MIS-C and sepsis patients questioned the utility of IL-6 in diagnosis and risk stratification by demonstrating much higher IL-6 levels in sepsis than in children with critical MIS-C (64).

Limitations of this investigation include the use of an immune gene panel, meaning that the role of non-immune genes could not be considered, the lack of a replication cohort, and the relatively small number of JSLE and KD patients. Further research may be required to determine whether IL-27 is upregulated in KD or KD shock syndrome. We were also unable to completely adjust associations for NLR due to the availability of these data for only a subset of the MIS-C cohort and none of the healthy controls, meaning estimated values were used for the healthy control cohort. Nevertheless, we were able to describe patterns of global gene dysregulation including up-regulation of genes involved in several pathways relevant to MIS-C pathogenesis, for the first time in a South African patient cohort. It should be noted that these results were limited to the RNA level, and validation at the protein and cytokine levels, although beyond the scope of this study, will be preferable in future.

Cumulatively, these findings support the role of innate immune pathways in the development of MIS-C with particular enrichment of IL-1 and pyroptosis gene expression in MIS-C. *IL27* was the only gene that could distinguish between MIS-C and other febrile conditions in our setting. As a potent anti-inflammatory and antiviral cytokine with documented links to COVID-19 and vaccine responses, this may be a promising cytokine in the pathogenesis or treatment of MIS-C that will warrant further investigation.

## Data availability statement

The datasets presented in this study can be found in online repositories. The names of the repository/repositories and accession number(s) can be found below: https://zivahub.uct.ac.za/, http://doi.org/10.25375/uct.20286243.

## Ethics statement

The studies involving human participants were reviewed and approved by Human Research Ethics Committee, Faculty of Health Sciences, University of Cape Town. Written informed consent to participate in this study was provided by the participants’ legal guardian/next of kin.

## Author contributions

KW designed and led the project. RNA expression data were generated by SCM, ME, RB and TJS and analysed by KW, GK, TFS and CB. TFS and KW prepared the first draft of the manuscript. HF-T, RS, DA, CB, CS, JD and TFS assisted with recruiting participants, gathering data and editing the manuscript. LJZ and CS provided institutional and intellectual support for the project and LJZ supplied funding with KW. All authors confirm that they had full access to all the data in the study and accept responsibility to submit for publication. All authors have read and approved the manuscript for submission.

## Funding

This work was supported by the Crick African Network (CAN), through a fellowship to KW. This fellowship funded Dr Webb’s salary and study consumables. The CAN receives its funding from the UK’s Global Challenges Research Fund (MR/P028071/1), and by the Francis Crick Institute which receives its core funding from Cancer Research UK (FC1001647), the UK Medical Research Council (FC1001647), and the Wellcome Trust (FC1001647). This work was also funded by a grant from the Wellcome Centre for Infectious Disease Research in Africa (CIDRI) through a rapid COVID grant to Prof Liesl Zühlke and Dr Kate Webb which allowed for the funding of members of the team salaries (HF-T, DA, CB, TFS) and study consumables, software and hardware. SCM is a recipient of PhD funding from the South African Medical Association (SAMA), the Fogarty International Center of the National Institutes of Health (NIH) under Award Number D43 TW010559, the Harry Crossley Clinical Research Fellowship, and the South African Medical Research Council (SAMRC) through its Division of Research Capacity Development under the SAMRC Clinician Researcher Programme. The content is solely the responsibility of the authors and does not necessarily represent the official views of SAMA, the NIH, the Harry Crossley Foundation, or the SAMRC. This research was supported by South African Medical Research Council with funds received from National Treasury. The content and findings reported/illustrated are the sole deduction, view and responsibility of the researcher and do not reflect the official position and sentiments of the SAMRC or National Treasury. The funders did not play a role in the data collection, analysis, or interpretation; trial design; patient recruitment; or any aspect pertinent to the study.

## Acknowledgments

Graeme Wilson (anaesthetics), Shazia Peer (ENT), Elizabeth Mayne (NICD), William Horsnell, Christopher Tinley (ophthalmology), Dirk von Delft (surgery), John Lazarus (urology), John Lawrenson, George Comitis, Mignon McCulloch, Peter Nourse, Ashton Coetzee, Kirsty Donald, Shamiel Salie, ICU staff, theatre staff and the paediatric general staff at Red Cross War Memorial Children’s Hospital.

## Conflict of interest

The authors declare that the research was conducted in the absence of any commercial or financial relationships that could be construed as a potential conflict of interest.

## Publisher’s note

All claims expressed in this article are solely those of the authors and do not necessarily represent those of their affiliated organizations, or those of the publisher, the editors and the reviewers. Any product that may be evaluated in this article, or claim that may be made by its manufacturer, is not guaranteed or endorsed by the publisher.
