# Supplementary material for: IL27 gene expression distinguishes multisystem inflammatory syndrome in children from febrile illness in a South African cohort
Source: Front Immunol. 2022 Sep 6;13:992022. doi: 10.3389/fimmu.2022.992022 (PMC9486543; doi:10.3389/fimmu.2022.992022)

Supplementary Material

**Supplementary table 1:** qRT-PCR primer-probe assay panel

| **Gene** | **TaqMan assay ID** | **Inclusion in analysis** |
| --- | --- | --- |
| Reference genes | | |
| *ACTR3* | Hs01029159_g1 | Included (passed QC) |
| *CDC42* | Hs03044122_g1 | Included (passed QC) |
| *USF2* | Hs01100994_g1 | Included (passed QC) |
| Genes of interest | | |
| *ALOX5* | Hs01095330_m1 | Included (passed QC) |
| *ALOX12* | Hs00167524_m1 | Included (passed QC) |
| *BPI* | Hs01552756_m1 | Included (passed QC) |
| *CAMP* (Cathelicidin) | Hs00189038_m1 | Included (passed QC) |
| *CASP1* | Hs00354836_m1 | Included (passed QC) |
| *CASP3* | Hs00234387_m1 | Included (passed QC) |
| *CASP5* | Hs00362078_m1 | Included (passed QC) |
| *CCL2* (MCP1) | Hs00234140_m1 | Included (passed QC) |
| *CCL4* (MIP1B) | Hs01031494_m1 | Included (passed QC) |
| *CCL5* | Hs00174575_m1 | Included (passed QC) |
| *CCL8* (MCP2) | Hs04187715_m1 | Included (passed QC) |
| *CD274* (PD-L1) | Hs01125299_m1 | Included (passed QC) |
| *CTLA4* | Hs03044418_m1 | Included (passed QC) |
| *CXCL8* (IL8) | Hs99999034_m1 | Included (passed QC) |
| *CXCL10* | Hs00171042_m1 | Included (passed QC) |
| *CXCR3* | Hs01847760_s1 | Included (passed QC) |
| *DDX58* | Hs00204833_m1 | Included (passed QC) |
| *DEFA1* | Hs00234383_m1 | Included (passed QC) |
| *DEFA4* | Hs01056651_g1 | Included (passed QC) |
| *FCGR1B* | HSS02341825_m1 | Included (passed QC) |
| *FCGR2A* | Hs01017702_g1 | Included (passed QC) |
| *FOXP3* | Hs01085834_m1 | Included (passed QC) |
| *GBP2* | HS00894846_g1 | Included (passed QC) |
| *GNLY* | Hs00246266_m1 | Included (passed QC) |
| *GZMA* | Hs00989184_m1 | Included (passed QC) |
| *GZMB* | Hs01554355_m1 | Included (passed QC) |
| *HNP3* | Hs00414018_m1 | Included (passed QC) |
| *IFI16* | Hs00194261_m1 | Included (passed QC) |
| *IFNAR1* (IFR) | Hs01066118_m1 | Included (passed QC) |
| *IFNAR2* (IFR2) | Hs01022060_m1 | Included (passed QC) |
| *IFNG* | Hs00989291_m1 | Included (passed QC) |
| *IFNGR1* | Hs00166223_m1 | Included (passed QC) |
| *IL10* | Hs00961622_m1 | Included (passed QC) |
| *IL12A* | Hs00168405_m1 | Included (passed QC) |
| *IL15* | Hs01003716_m1 | Included (passed QC) |
| *IL15RA* | Hs00233692_m1 | Included (passed QC) |
| *IL1B* | Hs01555410_m1 | Included (passed QC) |
| *IL27* | Hs00377366_m1 | Included (passed QC) |
| *IL32* | Hs00992441_m1 | Included (passed QC) |
| *IL6* | Hs00985639_m1 | Included (passed QC) |
| *IL7R* (ILRA) | Hs00893626_m1 | Included (passed QC) |
| *IRF1* | Hs00971960_m1 | Included (passed QC) |
| *IRF7* | Hs01014809_g1 | Included (passed QC) |
| *IRF8* | Hs01128710_m1 | Included (passed QC) |
| *IRGM* (IRGM1) | Hs01013699_s1 | Included (passed QC) |
| *ISG15* | Hs01921425_s1 | Included (passed QC) |
| *LRRC32* (GARP) | Hs01017468_m1 | Included (passed QC) |
| *LTA4H* | Hs01075871_m1 | Included (passed QC) |
| *LTF* | Hs00914334_m1 | Included (passed QC) |
| *MARCO* | Hs00198935_m1 | Included (passed QC) |
| *MMP14* | Hs01037009_g1 | Included (passed QC) |
| *MMP25* | Hs01554789_m1 | Included (passed QC) |
| *MMP8* | Hs01029057_m1 | Included (passed QC) |
| *MMP9* | Hs00957562_m1 | Included (passed QC) |
| *MX1* | Hs00895608_m1 | Included (passed QC) |
| *MX2* | Hs01550811_m1 | Included (passed QC) |
| *NOD2* | Hs00223394_m1 | Included (passed QC) |
| *OAS1* | Hs00973637_m1 | Included (passed QC) |
| *PPID* (Cyclophilin D) | Hs04193937_gH | Included (passed QC) |
| *PRF1* (Perforin) | Hs00169473_m1 | Included (passed QC) |
| *PTGER2* | Hs04183523_m1 | Included (passed QC) |
| *RAB33A* | Hs00191243_m1 | Included (passed QC) |
| *RPL13A* | Hs04194366_g1 | Included (passed QC) |
| *SDR39UI* | Hs01016970_g1 | Included (passed QC) |
| *SERPING1* | Hs00934329_m1 | Included (passed QC) |
| *SLAMF7* | Hs00221793_m1 | Included (passed QC) |
| *SOCS1* | Hs00705164_s1 | Included (passed QC) |
| *STAB1* | Hs01109068_m1 | Included (passed QC) |
| *STAT2* | Hs01013123_m1 | Included (passed QC) |
| *TGFB1* (TGFB) | Hs00998133_m1 | Included (passed QC) |
| *TIMP1* | Hs99999139_m1 | Included (passed QC) |
| *TIMP2* | Hs00234278_m1 | Included (passed QC) |
| *TLR8* | Hs00607866_mH | Included (passed QC) |
| *TNF* (TNFA) | Hs01113624_g1 | Included (passed QC) |
| *TNFR2* | Hs00961748_m1 | Included (passed QC) |
| *TNFRSF1A* (TNFR1) | Hs01042313_m1 | Included (passed QC) |
| *TNFRSF25* | Hs00237056_m1 | Included (passed QC) |
| *TREM1* | Hs00218624_m1 | Included (passed QC) |
| *TRMT2A* | Hs01000041_g1 | Included (passed QC) |
| *TUBGCP6* | Hs00363509_g1 | Included (passed QC) |
| *GMCSF* | Hs00929873_m1 | **Excluded (failed QC)** |
| *IL12B* | Hs01011519_m1 | **Excluded (failed QC)** |
| *IL22* | Hs01574154_m1 | **Excluded (failed QC)** |
| *MMP1* | Hs00899658_m1 | **Excluded (failed QC)** |
| *MMP2* | Hs01548727_m1 | **Excluded (failed QC)** |
| *PTGES2* | Hs00228159_m1 | **Excluded (failed QC)** |
| *VDR* | Hs00172113_m1 | **Excluded (failed QC)** |

QC – quality control

**Supplementary table 2:** Alternate diagnoses amongst the inflammatory control cohort

| **Alternate diagnosis** | **Frequency** |
| --- | --- |
| **Respiratory infections** | |
| Tuberculosis and/or pneumonia and/or SARS-CoV-2 | 4 |
| Unspecified viral lower tract respiratory infection | 1 |
| **Other infections** | |
| Typhoid | 1 |
| Suspected skin infection | 1 |
| Dysentery | 1 |
| COVID-exposed UTI | 1 |
| **Systemic disorders with or without viral infection** | |
| Henoch-Schonlein purpura | 1 |
| DiGeorge syndrome with viral infection | 1 |
| Systemic juvenile idiopathic arthritis with macrophage activation syndrome | 1 |
| Kikuchi syndrome | 1 |
| **Abdominal inflammation and/or infection** | |
| Perforated appendix | 1 |
| Appendicitis | 1 |
| COVID-exposed abdominal sepsis | 1 |
| Abdominal sepsis | 1 |
| **Other** | |
| Unspecified | 2 |

**Supplementary table 3:** Clinical presentation and outcomes of the MIS-C qPCR cohort

| **Characteristic** | **MIS-C cohort (n = 30)** | **Inflammatory controls (n = 19)** |
| --- | --- | --- |
| **Presenting symptom** | | |
| Fever | 30 (100.0%) | 16 (84.2%) |
| Tachycardia | 30 (100.0%) | 10 (52.6%) |
| Rash | 28 (93.3%) | 7 (36.8%) |
| Conjunctivitis | 21 (70.0%) | 1 (5.3%) |
| Abdominal pain | 17 (56.7%) | 11 (57.9%) |
| Diarrhoea | 13 (43.3%) | 8 (42.1%) |
| Arthritis | 13 (43.3%) | 5 (26.3%) |
| CNS disease | 8 (26.7%) | 2 (10.5%) |
| Headache | 8 (26.7%) | 4 (21.1%) |
| Lung disease | 6 (20.0%) | 4 (21.1%) |
| Renal disease | 3 (10.0%) | 2 (10.5%) |
| **SARS-CoV-2 test** | | |
| PCR positive (%) | 3 (10.0%) | 1 (5.3%) |
| Antibody positive (%) | 30 (100.0%) | 7 (36.8%) |
| **In-hospital treatment** | | |
| Oxygen | 11 (36.7%) | 1 (5.3%) |
| Ionotropes | 9 (30.0%) | 2 (10.5%) |
| Ventilation | 2 (6.7%) | 2 (10.5%) |
| Dialysis | 2 (6.7%) | 0 |
| ICU | 9 (30.0%) | 2 (10.5%) |
| Median hospital stay | 7.0 days | 9.1 days |

**Supplementary table 4:** Differentially expressed genes in MIS-C compared to healthy controls

| **Gene** | **Mean expression fold change** | **Unadjusted p-value** |
| --- | --- | --- |
| *CXCR3* | 0.320276272 | 2.90E-11 |
| *IL10* | 8.855476688 | 4.21E-11 |
| *IL27* | 7.107176288 | 6.04E-11 |
| *FCGR1B* | 4.629779818 | 7.00E-11 |
| *MMP8* | 141.267966 | 3.65E-10 |
| *TLR8* | 3.38930621 | 8.46E-10 |
| *CD274* | 7.643936854 | 9.42E-10 |
| *CASP5* | 4.337386053 | 2.53E-09 |
| *BPI* | 12.95296231 | 2.60E-09 |
| *RAB33A* | 0.53924239 | 1.86E-08 |
| *IL1B* | 2.535659396 | 3.90E-08 |
| *MMP25* | 2.805635737 | 1.70E-07 |
| *GZMB* | 0.533401473 | 2.30E-07 |
| *TIMP2* | 2.415364663 | 2.58E-07 |
| *GZMA* | 0.389303948 | 3.01E-07 |
| *TNFRSF25* | 0.591044431 | 3.54E-07 |
| *PRF1* | 0.536808956 | 1.91E-06 |
| *TRMT2A* | 0.597838623 | 1.97E-06 |
| *TUBGCP6* | 0.643440741 | 2.57E-06 |
| *SDR39U1* | 0.644552619 | 4.47E-06 |
| *IL7R* | 2.754169418 | 7.00E-06 |
| *FCGR2A* | 4.021941726 | 1.03E-05 |
| *CASP1* | 1.581755606 | 1.06E-05 |
| *FOXP3* | 0.660831412 | 2.12E-05 |
| *DEFA1* | 11.45380406 | 2.23E-05 |
| *MCP1* | 0.662317603 | 4.49E-05 |
| *CAMP* | 3.091494067 | 5.49E-05 |
| *MMP9* | 9.91862501 | 5.56E-05 |
| *IFNAR1* | 1.476729883 | 7.00E-06 |

**Supplementary table 5:** Associations of gene expression with MIS-C after adjustment for neutrophil-lymphocyte ratio

| **Gene** | **Mean expression fold change** | **Adjusted p-value*** |
| --- | --- | --- |
| *FCGR1B* | 4.629779818 | **0.004553** |
| *IL10* | 8.855476688 | **0.00688** |
| *CD274* | 7.643936854 | **0.00849** |
| *BPI* | 12.95296231 | **0.013704** |
| *IL1B* | 2.535659396 | **0.01637** |
| *CAMP* | 3.091494067 | **0.0190** |
| *MMP8* | 141.267966 | **0.02460** |
| *IL27* | 7.107176288 | **0.02983** |
| *CXCR3* | 0.320276272 | **0.0325** |
| *DEFA1* | 11.45380406 | **0.0389** |
| *MMP9* | 9.91862501 | **0.03937** |
| *SDR39U1* | 0.644552619 | 0.0533 |
| *CASP1* | 1.581755606 | 0.054593 |
| *TLR8* | 3.38930621 | 0.0745 |
| *GZMB* | 0.533401473 | 0.158 |
| *MMP25* | 2.805635737 | 0.16027 |
| *CASP5* | 4.337386053 | 0.16268 |
| *FOXP3* | 0.660831412 | 0.3250 |
| *TIMP2* | 2.415364663 | 0.41489 |
| *MCP1* | 0.662317603 | 0.5763 |
| *FCGR2A* | 4.021941726 | 0.63660 |
| *TRMT2A* | 0.597838623 | 0.6438 |
| *RAB33A* | 0.53924239 | 0.7381 |
| *PRF1* | 0.536808956 | 0.7716 |
| *IL7R* | 2.754169418 | 0.8024 |
| *IFNAR1* | 1.476729883 | 0.8024 |
| *TUBGCP6* | 0.643440741 | 0.9103 |
| *GZMA* | 0.389303948 | 0.97917 |
| *TNFRSF25* | 0.591044431 | 0.9861 |

* P-values after adjusting for neutrophil/lymphocyte ratio using binomial logistic regression for 17 MIS-C patients and 54 estimated values for healthy controls

**Supplementary table 6:** Pairwise comparison of MIS-C and other febrile conditions

| **Gene** | **P-value** | **Significant after Holm correction** |
| --- | --- | --- |
| *IL27* | 0.00056 | Yes |
| *CD274* | 0.00447 | Not significant |
| *ILRA* | 0.0052 | Not significant |
| *IL1B* | 0.00523 | Not significant |
| *BPI* | 0.0061 | Not significant |
| *IL10* | 0.00654 | Not significant |
| *CASP5* | 0.00677 | Not significant |
| *RAB33A* | 0.00784 | Not significant |
| *PTGER2* | 0.00815 | Not significant |
| *SOCS1* | 0.0095 | Not significant |
| *CXCR3* | 0.01158 | Not significant |
| *TLR8* | 0.01245 | Not significant |
| *FoxP3* | 0.01682 | Not significant |
| *TIMP2* | 0.02256 | Not significant |
| *IL15RA* | 0.02438 | Not significant |
| *TNFR1* | 0.02482 | Not significant |
| *MCP2* | 0.02938 | Not significant |
| *TUBGCP6* | 0.03281 | Not significant |
| *IL15* | 0.03404 | Not significant |
| *DEFA4* | 0.03462 | Not significant |
| *TRMT2A* | 0.03462 | Not significant |
| *MMP25* | 0.04909 | Not significant |
| *LTA4H* | 0.05007 | Not significant |
| *MIP1B* | 0.05772 | Not significant |
| *GARP* | 0.06226 | Not significant |
| *TNFR2* | 0.06477 | Not significant |
| *IL6* | 0.06495 | Not significant |
| *CASP1* | 0.06673 | Not significant |
| *MMP9* | 0.07366 | Not significant |
| *OAS1* | 0.085 | Not significant |
| *ISG15* | 0.08883 | Not significant |
| *MX2* | 0.089 | Not significant |
| *IFI16* | 0.09863 | Not significant |
| *DEFA1* | 0.11161 | Not significant |
| *ALOX12* | 0.1122 | Not significant |
| *FCGR1B* | 0.11942 | Not significant |
| *FCGR2A* | 0.11975 | Not significant |
| *IL8* | 0.12022 | Not significant |
| *TNFRSF25* | 0.12261 | Not significant |
| *IL12A* | 0.12456 | Not significant |
| *IFNG* | 0.13081 | Not significant |
| *IRGM1* | 0.15163 | Not significant |
| *IFNGR1* | 0.15581 | Not significant |
| *IFR* | 0.16092 | Not significant |
| *GNLY* | 0.17124 | Not significant |
| *HNP3* | 0.1959 | Not significant |
| *IRF7* | 0.2057 | Not significant |
| *MARCO* | 0.21675 | Not significant |
| *MCP1* | 0.23301 | Not significant |
| *STAT2* | 0.23485 | Not significant |
| *SDR39U1* | 0.23531 | Not significant |
| *TGFB* | 0.2444 | Not significant |
| *MMP14* | 0.24811 | Not significant |
| *GZMA* | 0.26522 | Not significant |
| *CyclophilinD* | 0.30059 | Not significant |
| *MMP1* | 0.30245 | Not significant |
| *MMP8* | 0.30595 | Not significant |
| *GZMB* | 0.3069 | Not significant |
| *STAB1* | 0.31274 | Not significant |
| *TREM1* | 0.33175 | Not significant |
| *NOD2* | 0.34532 | Not significant |
| *GBP2* | 0.38545 | Not significant |
| *DDX58* | 0.41254 | Not significant |
| *IRF1* | 0.44516 | Not significant |
| *IFR2* | 0.47586 | Not significant |
| *IL32* | 0.48027 | Not significant |
| *SERPING1* | 0.48273 | Not significant |
| *Perforin* | 0.51667 | Not significant |
| *CCL5* | 0.53324 | Not significant |
| *TIMP1* | 0.54886 | Not significant |
| *LTF* | 0.60751 | Not significant |
| *MX1* | 0.63399 | Not significant |
| *TNFA* | 0.77351 | Not significant |
| *CXCL10* | 0.79258 | Not significant |
| *CASP3* | 0.80844 | Not significant |
| *ALOX5* | 0.81045 | Not significant |
| *CTLA4* | 0.84965 | Not significant |
| *IRF8* | 0.87025 | Not significant |
| *RPL13A* | 0.95243 | Not significant |
| *Cathelicidin* | 0.9935 | Not significant |


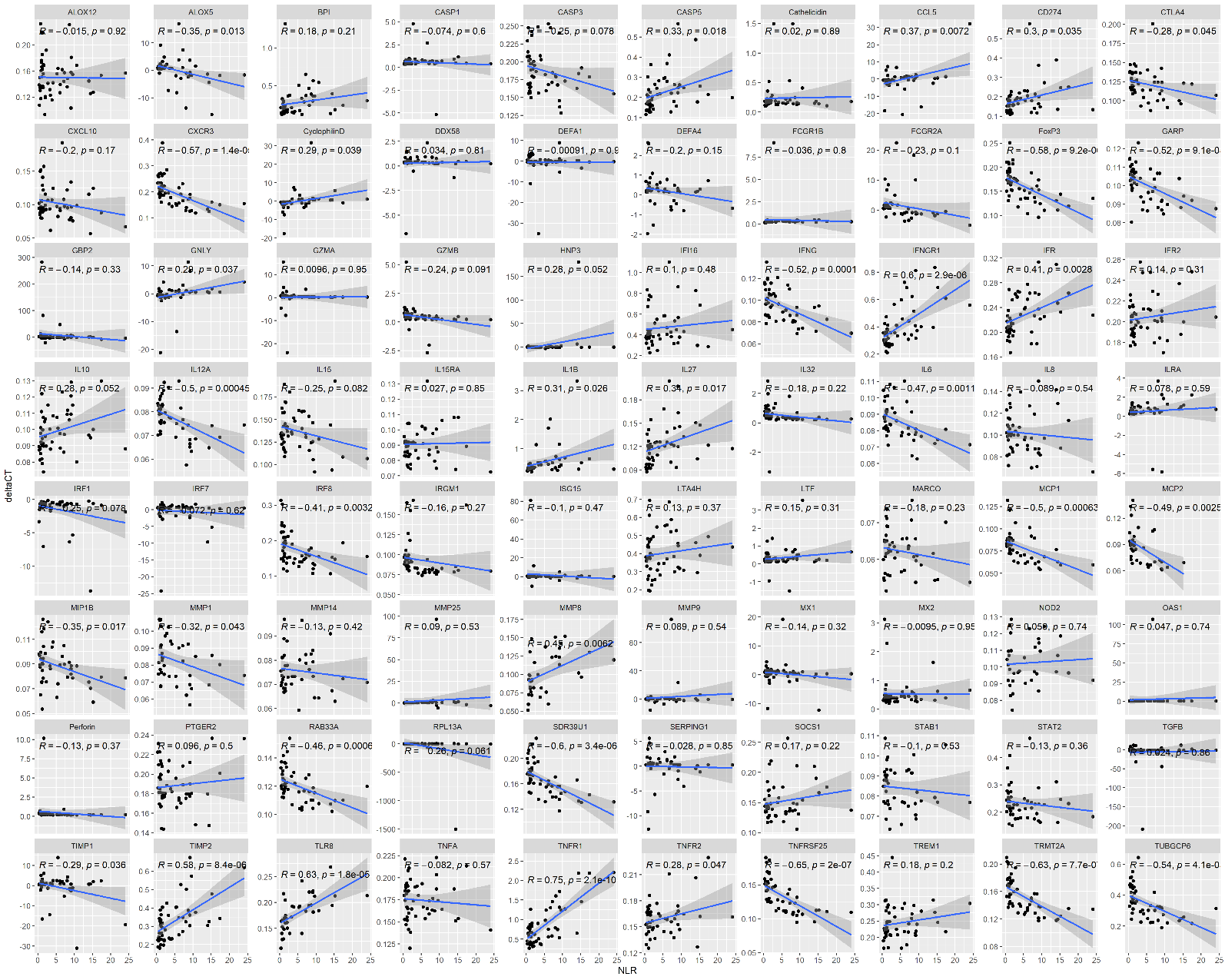


**Supplementary Figure 1.** Linear relationships between neutrophil-lymphocyte ratio and gene expression for each gene of interest.


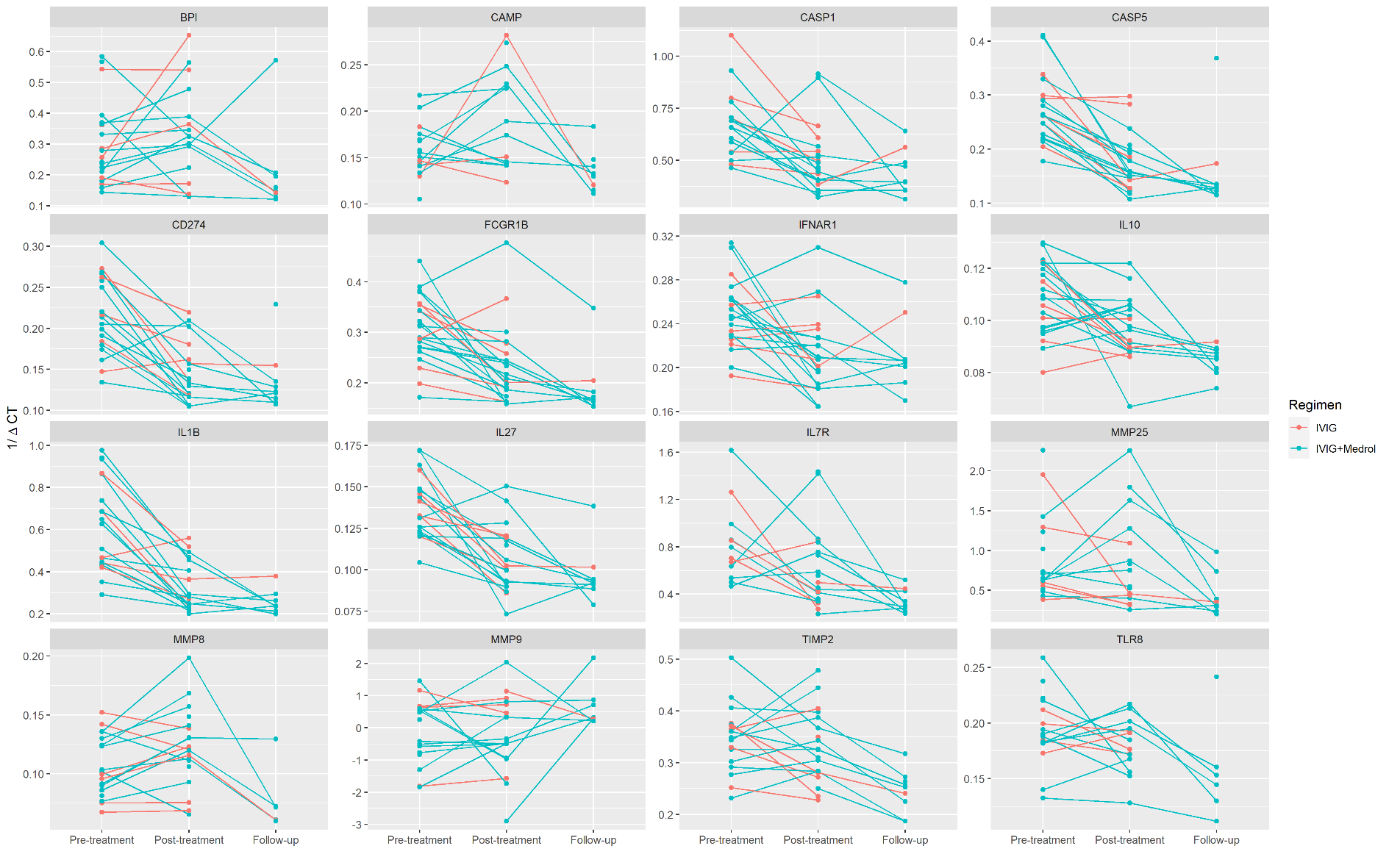


**Supplementary Figure 2.** Analysis of pre- and post-treatment gene expression of differentially up-regulated genes in MIS-C patients.


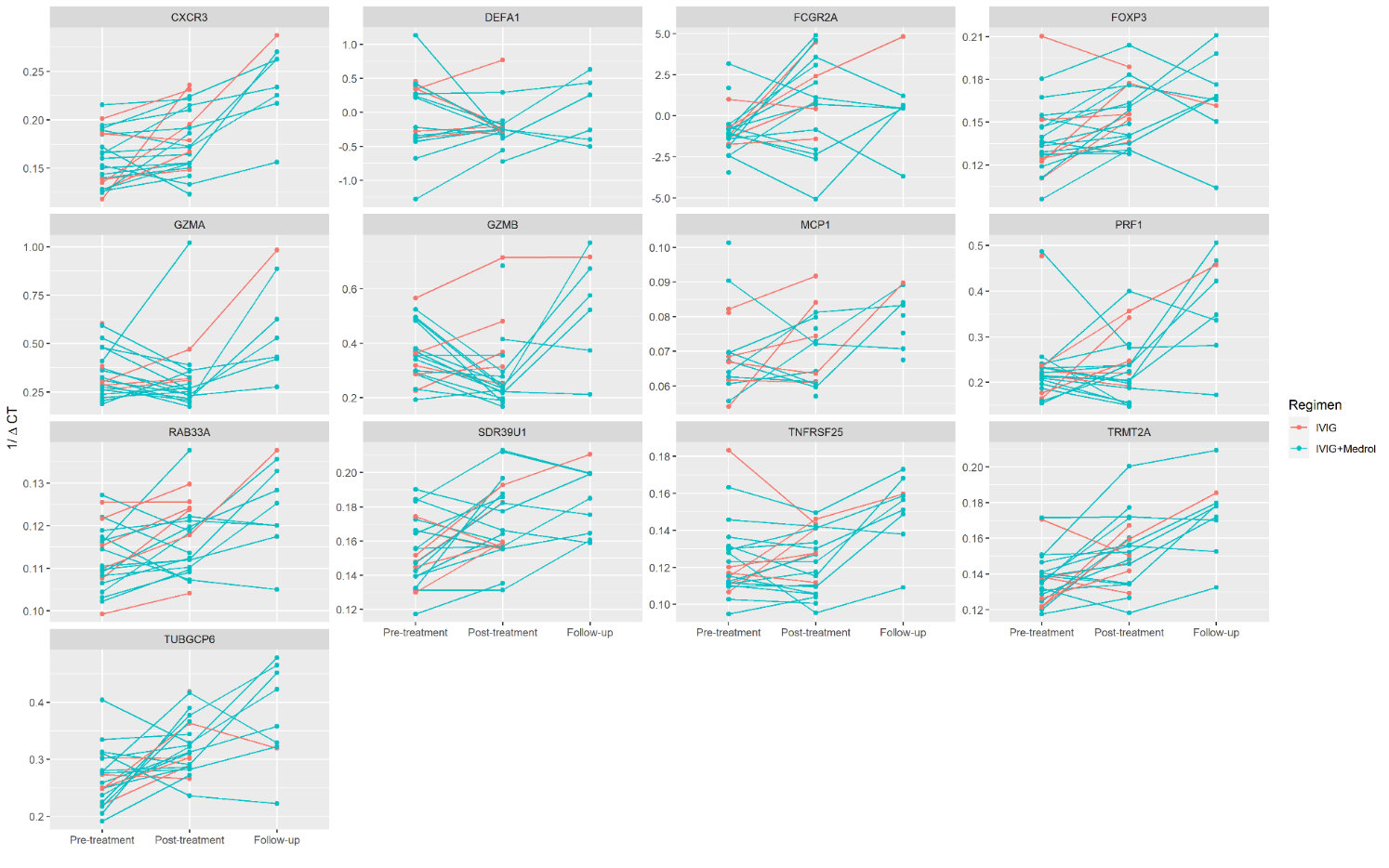


**Supplementary Figure 3.** Analysis of pre- and post-treatment gene expression of differentially down-regulated genes in MIS-C patients.


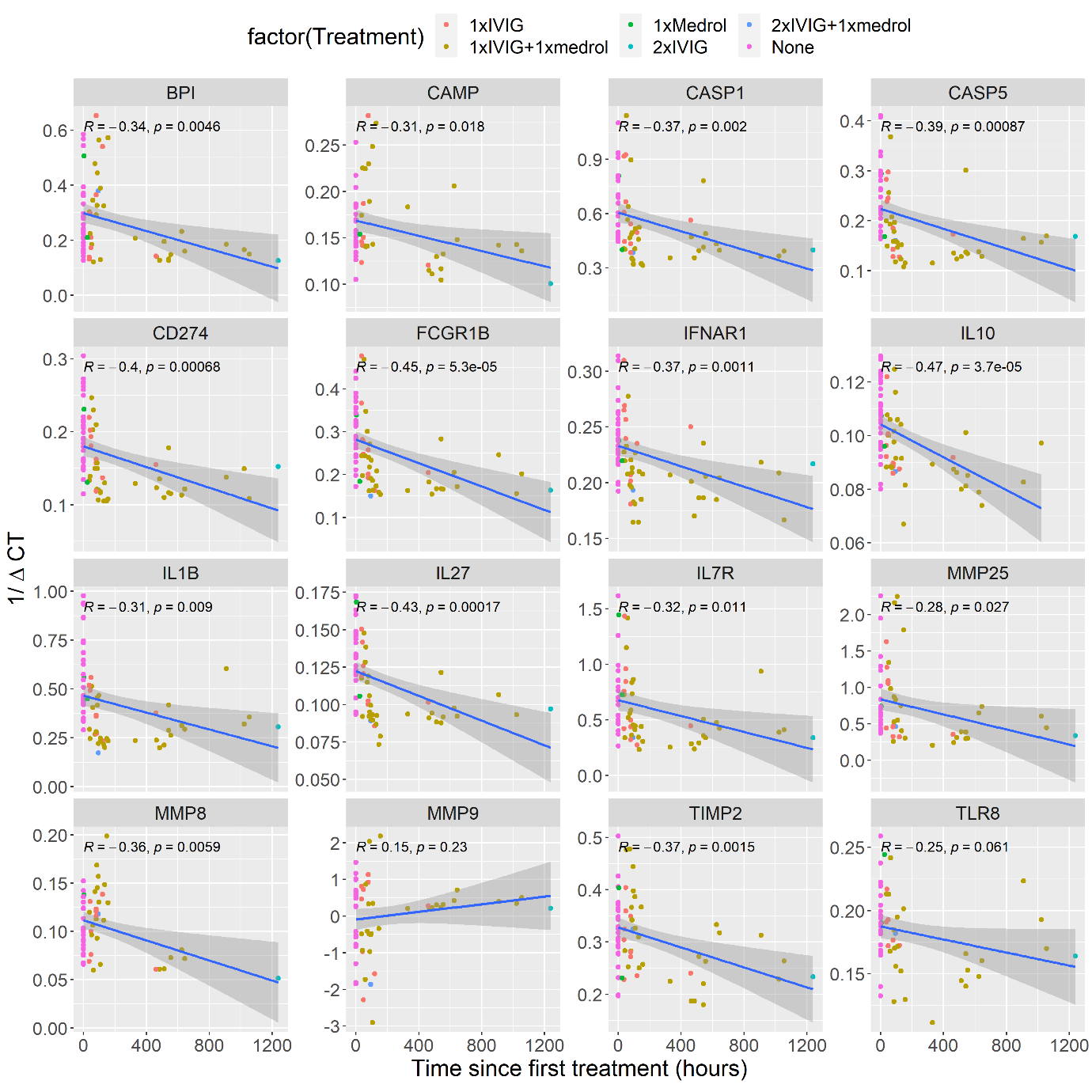


**Supplementary Figure 4.** Scatterplot of up-regulated gene expression over time since first treatment.


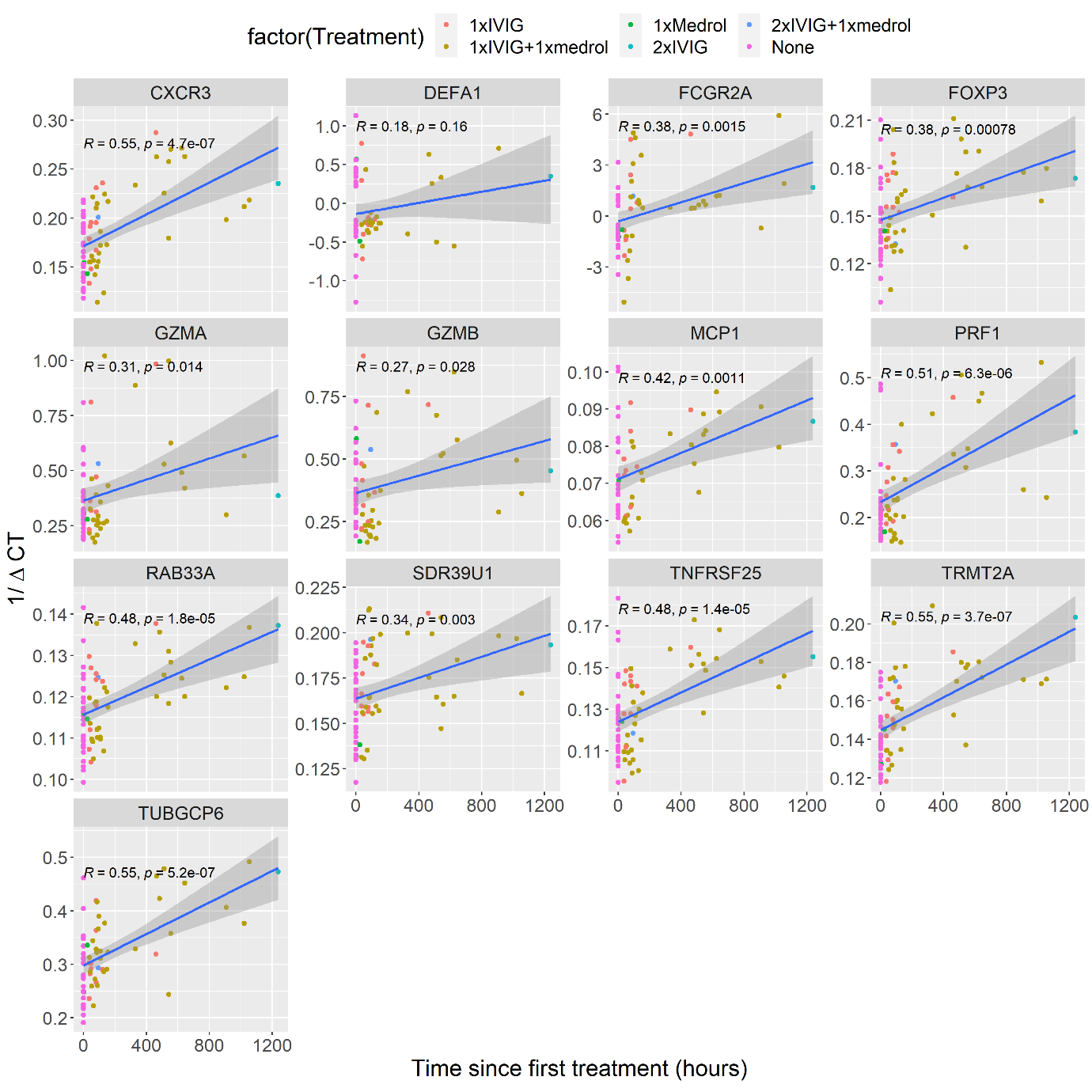


**Supplementary Figure 5.** Scatterplot of down-regulated gene expression over time since first treatment.

**A**


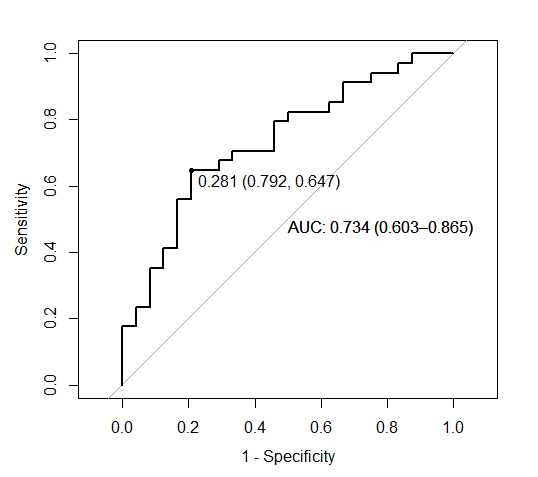


**B**


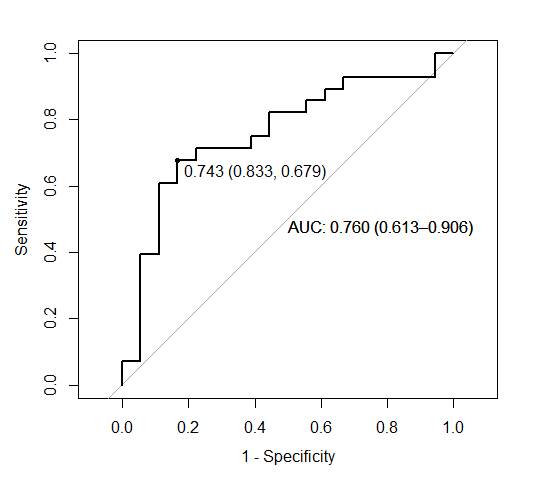


**Supplementary figure 6:** ROC analysis of gene expression scores based on pairwise comparison of MIS-C to other febrile conditions. (A) *IL27* + *CD274*; (B) *IL27* + *CD274* + *IL7R* (C) *IL27* + *CD274* + *IL7R* + *IL1B*; (D) *IL27* + *CD274* + *IL7R* + *IL1B* + *BPI*

**C**


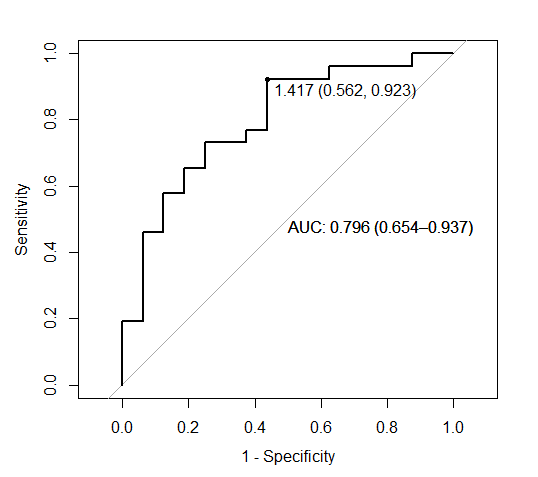


**D**


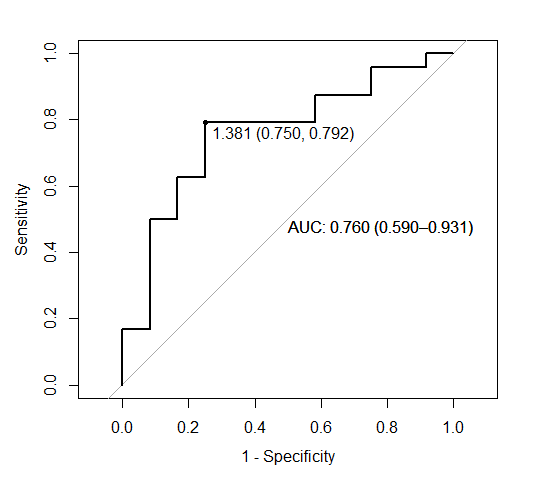

Supplement: Supplementary file 1 [file DataSheet_1.docx]
